# Assessing the level of institutionalization of donor-funded anti-retroviral therapy (ART) programs in health facilities in Uganda: implications for program sustainability

**DOI:** 10.1080/16549716.2018.1523302

**Published:** 2018-10-08

**Authors:** Henry Zakumumpa, Japheth Kwiringira, Joseph Rujumba, Freddie Ssengooba

**Affiliations:** a School of Public Health, Makerere University, Kampala, Uganda; b Faculty of Social Sciences, Kyambogo University, Kampala, Uganda; c School of Medicine, Makerere University, Kampala, Uganda

**Keywords:** Health systems, sustainability, human resources for health, HIV, case-study, health services, program evaluation

## Abstract

**Background**: In the context of declining international assistance for ART scale-up in Sub-Saharan Africa, the institutionalization of ART programs through integrating them in the organizational routines of health facilities is gaining importance as a program sustainability strategy.

**Objective**: The aims of this study were; (i) to compare the level of institutionalization of ART programs in health facilities in Uganda and (ii) to explore reasons for variations in the degree of program institutionalization.

**Methods**: In Phase One, we utilized Level of Institutionalization Scales developed by Goodman (1993) to measure the degree of institutionalization of ART interventions in 195 health facilities across Uganda. The 45-item questionnaire measured institutionalization based on four sub-systems (production, maintenance, supportive, managerial) theorized to make up an organization assessed against two levels of institutionalization; routines (lowest) niche saturation (highest). In Phase Two, four health facilities were purposively selected (2 with the highest and 2 with the lowest institutionalization scores) for a multiple case-study involving semi-structured interviews with ART clinic managers(n = 32), on-site observations and document review.

**Results**: The two highest scoring health facilities had a longer HIV intervention implementation history of between 8 and 11 years. The highest scoring cases associated intervention institutionalization with sustained workforce trainings in ART management, the retention of ART-trained personnel and generating in-house ART manuals. The turnover of ART-proficient staff was identified as a barrier to intervention institutionalization in the lowest-ranked cases. Significant differences in organizational contexts were identified. The two highest-ranked health facilities were well-established, higher-tier hospitals while the lowest scoring health facilities were lower-level health facilities.

**Conclusions**: The level of institutionalization of ART interventions appeared to be differentiated by level of care in the Ugandan health system. Interventions aimed at strengthening program institutionalization in lower-level health centers at the level of human resources for health could enhance ART scale-up sustainability.

## Background

For over a decade, the calls for universal access to anti-retroviral therapy (ART) have been gaining momentum in the global health agenda [1]. In 2014, UNAIDS unveiled the ambitious 90-90-90 targets a part of which aims at enrolling 90% of those with HIV on sustained ART by 2020 [2]. In November 2015, WHO released new global ART treatment guidelines recommending that all diagnosed with HIV be enrolled on sustained ART regardless of disease stage [3,4].

Out of the 37 million living with HIV globally, only 18 million were enrolled on ART by mid-end of 2016 [5]. In Sub-Saharan Africa (SSA), the region with the highest HIV burden in the world [6], the accelerated expansion in ART coverage, has depended substantially on global health initiatives (GHIs) principally, The President’s Emergency Fund for AIDS Relief (PEPFAR) and The Global Fund [7]. South Africa, Nigeria and Uganda account for nearly half of all new HIV infections in SSA [8]. In Uganda, an estimated 85% of the national HIV response is externally funded principally through PEPFAR and The Global Fund which have supported ART scale-up since June 2004 [9,10]. National ART roll-out commenced at a relatively higher-level of care starting at major tertiary hospitals in Uganda [4]. ART services were subsequently decentralized to lower-level health facilities including in small for-profit clinics [4]. The goal of ART scale-up was to expand coverage through commissioning multiple sites with the aim of extending service delivery beyond large tertiary hospitals [9]. It is therefore imperative to assess the degree of institutionalization of ART interventions in lower-level health facilities in order to determine long-term program sustainability outcomes in primary care health facilities and to inform further ART scale-up efforts.

International assistance for ART scale-up in Uganda was been characterized by a strong vertical approach. Indeed, a substantial component of this donor support was not integrated into the mainstream Ugandan health system [1]. During the ART scale-up phase, health facilities received a package of support comprising; a free supply of HIV commodities, health worker training in ART management, on-site support supervision and the strengthening of ART program reporting [10].

There are persistent reports of a decline in international assistance for further ART scale-up in SSA [7,9]. Against this background, the sustainability of the implementation of ART scale-up in SSA has come into critical focus [1,4,9–11]. The institutionalization or integration of interventions in the organizational routines of health service organizations is considered an important indicator of long-term program sustainability [12–16]. Although there have been numerous studies documenting the initial phase of ART scale-up in SSA [4,7], there is a dearth of systematic analysis of level of institutionalization of ART programs in externally supported health facilities in Uganda and the broader Sub-Saharan Africa region.

Institutionalization is theorized to occur when interventions become a habitual, routine and integral part of ‘normal’ health service delivery which is regarded as an important dimension of long-term program sustainability [13–16]. ‘In this perspective, the maintenance of program activities without special external funding is most likely to occur if the program components become embedded into organizational processes’’ [14]. As such, eliciting best practices from organizations with high degrees of institutionalization could enhance our level of understanding of the organizational attributes impacting on the institutionalization of ART programs and the prerequisites for achieving program sustainability [17–20]. The objectives of this study were to compare the level of institutionalization of ART programs in four health facilities in Uganda and to explore reasons for variations in the degree of program institutionalization.

## Methods

### Research design

This paper presents results from the second part of a broader two-phased mixed-methods study assessing the extent of institutionalization of ART interventions in 195 health facilities across Uganda [21,22,23]. We report here findings from the second qualitative phase which adopted a case-study research design. Yin [24] recommends case-study designs for in-depth investigation of organizations and their practices. Data were collected between February and June 2015.

### Study sites and sample selection

Four health facilities were purposively selected from a nationally representative sample of 195 health across Uganda (by geographic sub-region, ownership-type and level of care) which were enrolled in a survey to measure the degree of institutionalization of ART programs in an earlier study phase [21].The 195 health facilities participated in an externally funded emergency ART roll-out phase in Uganda between 2004 and 2009 [25].

The results from the survey (phase 1 of the study) [21] were used to select health facilities for in-depth study (phase 2). Two health facilities with the highest score for the institutionalization for ART programs and two health facilities with the lowest scores, in our national sample of 195 health facilities were purposefully selected for in-depth study.

The outcome of the selection of the four health facilities resulted in an appropriate mix in the study sample with regard to; (a) Setting (rural/urban) (b) ownership-type (Public, for-profit, not-for-profit) (c) level of care in the Ugandan health system [26] (Hospitals, health centers).

All four selected health facilities were accredited ART sites by Uganda’s Ministry of Health [27] suggesting the presence of a minimum level of facility infrastructure and service delivery capacity.

### Data collection

#### Phase I

##### Level of Institutionalization (LoIn) questionnaire

Adapted Level of Institutionalization (LoIn) scales developed by Goodman and colleagues [28] and subsequently tested for validity and reliability by Barab and colleagues [29] were used to measure the degree of institutionalization of ART programs in 195 health facilities across Uganda. The 45-item questionnaire measured the degree of institutionalization (routines, niche saturation) across four sub-systems that are theorized to make up a health facility [30]; (i) Production (ii) Maintenance, (iii) Managerial (iv) Supportive. Production is concerned with the procedures and mechanisms of delivering an intervention (e.g. if processes are laid out in manuals or standard operating procedures (SOPs). Maintenance is concerned with aspects relating to human resources management such as recruitment and sanction of personnel e.g. if permanent staff have been appointed to deliver the intervention. Supportive relates to the availability of organizational resources to facilitate implementation of the intervention such as if finances are made permanently available to support delivery of the intervention. Managerial is the sub-system charged with coordinating, directing and controlling all the other three sub-systems e.g. if monitoring and evaluation systems are in place. Institutionalization was assessed based on two levels; (i) routinization (a state where interventions progress to a habitual, routine part of normal service delivery and (ii) niche saturation (the highest possible expansion of the intervention in the organization’s ‘sub-systems’) [28]. During on-site visits by two investigators (HZ, FS), a hard-copy questionnaire was delivered at each of the four health facilities for purposes of being self-administered by the head of the ART clinic. A filled questionnaire was picked, on-site, by a research assistant one week from the initial visit by the investigators.

#### Phase II

##### Semi-structured interviews

A semi-structured interview guide consisting of structured questions (e.g. Position in ART clinic, length of service in ART clinic) and open-ended questions was developed. An example of the open-ended questions asked include *i) what factors facilitated or hindered the institutionalization of ART programs at your health facility? ii) How have you institutionalized ART delivery procedures in your health facility?*

Semi-structured interviews were conducted in English with a total of 12 participants with 3 representatives from each of the four health facilities. Individual, face-to-face, interviews were conducted by the first author with the head of the ART clinic (1) and the longest-serving ART clinicians (2) at each of the four health facilities.

Typically, the interviews lasted between 40–60 minutes and were conducted in the interviewees’ offices within the ART clinics. The interviews were audio recorded.

### On-site visit check list

An on-site visit checklist was developed for use during field visits by investigators. The checklist sought to verify if ART delivery procedures could be visually discerned by the investigator (e.g. ART delivery procedures pinned on staff noticeboards, written ART in-house manuals, pictorial guidance on class of ART medicines to be prescribed). Multiple visits were made to each of the four case-study facilities over the course of 12-weeks in the first half of 2015. Investigators spent at least 2–3 weeks at each of the case-study facilities including in engaging in informal discussions with the heads of the ART Clinic and the longest serving clinicians.

### Document review

Documentary analysis of relevant information relating to the case-study facilities was scrutinized (e.g. donor program evaluation reports, annual reports and strategic plans, web sites) to aid in constructing case descriptions (Section A) and to augment interviewee data (Section C).

### Data analysis

#### Phase I

##### The LoIn questionnaire

A summative score was determined for each of the four health facilities. The score was computed utilizing adapted Level of Institutionalization (LoIn) scales operationalized as an eight-factor model (Table 1) [31]. The degree of institutionalization was measured on two levels; (i) Routines and (ii) Niche saturation assessed across four sub-systems (Production, Managerial, Maintenance and Supportive). In total, there were eight domains (Table 1), with four representing routines and four representing niche saturation. A 4-point Likert scale was used for scoring both the routine and niche saturation components. For instance, for niche saturation items, a score of 1–4 was assigned (1 = No niche saturation, 2 = Minimum niche saturation, 3 = Moderate Niche saturation, 4 = Maximum niche saturation). Out of a maximum score of 32, a score was determined for each of the four case-study health facilities. The detailed scoring criteria is described here [28,31]. Data were managed in STATA (v. 13).

**Table 1.** Eight-factor model.

Dimensions (Extensiveness)	Degrees (Intensiveness)	
Organizational sub-systems	Routines	Niche saturation
PRODUCTION		
MAINTENANCE		
SUPPORTIVE		
MANAGERIAL		

Modified from: Goodman, R.M., McLeroy, K.R., Steckler, A.B., and Hoyle, R.H. (1993). ‘Development of Level of Institutionalization Scales for Health Promotion Programs.’ *Health Education Quarterly*, 20(2).

#### Phase II

##### Case-study analysis

We followed the processes for ensuring rigor in case-study analysis suggested by Gilson et al. [32] (See Table 2). To this end, the case-study analysis was conducted in four stages:

**Table 2.** Processes for ensuring rigour in case-study analysis adapted from Gilson et al (2012) [32].

PRINCIPLE	
Prolonged engagement	We spent 2–3 weeks at each of the four case-study facilities. Multiple on-site visits were spent engaging in informal discussions with clinicians and ART clinic managers, conducting formal, face-to-face interviews with multiple informants, filling out an on-site checklist based on observations and reading literature availed by health facilities relating to their ART programs such as annual reports and donor program evaluation reports.
Use of theory	Level of Institutionalization (LoIn) Scales developed by Goodman et al (1993) guided the study [28]. This framework is based on organizational theory and builds upon earlier work by Katz and Kahn (1978) ‘The Social psychology of organizations’ [30].
Case selection	Four health facilities were purposefully selected from a nationally representative sample of 195 health facilities across Uganda which were enrolled in a survey to measure the level of ART program Institutionalization. The two health facilities with highest scores and the two with the lowest scores in this national sample were selected.
Sampling	We aimed to have a sample that had appropriate representation of health facility demographics in Uganda with respect to a) setting(rural/urban), b) ownership-type(public, for-profit, not-for-profit)c) Level of care(tertiary, secondary, primary).
Multiple methods	Multiple methods were used including face-to-face interviews, a Level of Institutionalization questionnaire, on-site observation check lists, document review and informal engagements with clinicians and the head of the ART Clinic
Triangulation	Case descriptions were constructed based on triangulation across multiple data sources (Questionnaire data, interviewee data and document review).
Negative case analysis	Emergent themes that contradicted initial assumptions and the theory underpinning the study were identified.
Peer debriefing and support	Data analysis at each of the four stages (case description, with-in case, across-case, respondent validation) involved a team-based process involving at least three authors (HZ, JK, RJ). Across-case analyses were agreed upon by consensus involving all authors (HZ, SB, JK, RJ, FS).
Respondent validation	Initial cross-case analyses were presented to five selected respondents from each of the four case-study facilities for their review and comments (clinicians and ART Clinic managers). Their feedback informed final analyses.

#### Case description

In the first stage, an initial case description was constructed for each of the four case-study facilities by the first author based on a synthesis of data from multiple sources (LoIn questionnaire, provider interviewees, on-site visits and document review). Two authors (JK, JR) then reviewed the four preliminary case descriptions generated by the first author to assess accuracy and consistency with data contained in the LoIn questionnaire, provider interviews, on-site visits and document review.

#### Within-case analysis

In the second stage, preliminary within-case analyses were conducted by the first author. Both inductive and deductive analysis approaches were utilized to explore facilitators and barriers to the institutionalization of ART programs for each individual case. Deductive analysis was guided by the four domains (Production, Managerial, Maintenance and Supportive) in the theoretical framework adopted for the study [28]. The initial within-case analyses generated by the first author were further reviewed and discussed by three co-authors (JK, JR, HZ) through a consensus process.

#### Across-case analysis

The third stage involved across-case comparisons by exploring patterns of convergence or divergence of results across the two categories of cases; (a) P-001 and PNFP-001, b) P-002 and PFP-001. For ease of across-case analyses, a four-column table was constructed [33]. The table contained a summary of findings for each case generated from the within-case analysis phase. Across-case analyses aimed to identify differences and similarities in experiences across the four health facilities were performed in a team-based process involving all authors.

#### Respondent validation

In the fourth stage, a respondent data validation workshop [32] was conducted involving five respondents (facility in-charge (1), head of ART clinic (1), ART clinician (3)) from each of the four case-study facilities. The aim of the workshop was to garner feedback with respect to (i) the case-description constructed for each case-study facility and (ii) the cross-case analysis findings. Feedback from participants in the data validation workshop was incorporated in the final analyses.

## Results

The results emerging from this study are presented in three sections (Sections A, B and C).

Section A presents a description of the organizational characteristics of the four case-study facilities.

In section B, a selection of attributes from Level of institutionalization (LoIn) data, with respect to the four health facilities, is presented.

Section C presents findings from a qualitative analysis of facilitators and barriers to the institutionalization of ART programs at case-study facilities.

### Section A

#### Cases description

##### P-001

P-001 is a public, tertiary hospital in South-Western Uganda. The ART clinic is a specialized unit with a large hospital complex. The scale-up of ART services commenced in 2005. However, for this hospital, the earliest HIV intervention implementation history dates back to 1998. At the time of data collection, active patients enrolled on ART clinic were 24,407. The hospital provides both adult and pediatric ART services.

##### PNFP-001

PNFP-001 is a large private not-for-profit hospital based in the Ugandan capital, Kampala. It is categorized as a General Hospital by the Uganda government although it also serves a referral function. HIV services started at this hospital in 1998 with a handful of patients. ART scale-up at this hospital was first implemented in 2005. The hospital operates a vertical ART clinic within a large hospital complex. A number of patients active on ART number were 4,337. This hospital offers both adult and pediatric ART services.

##### P-002

P-002 is a public health centre IV or a facility serving an area equivalent to a sub-district or county [26]. This health facility is located in a rural setting in East Central Uganda. ART services at this facility commenced in 2006. The ART clinic has semi-permanent physical infrastructure such as a patient shade. The ART clinic runs once every week on Thursdays. The number of patients active on ART was 458. The clinic offers both adult and pediatric ART services.

##### PFP-001

PFP-001 is a for-profit clinic located in a peri-urban setting of central Uganda. There is no specialized ART clinic. HIV services are integrated with other health services. HIV patients queue for services with any other patients. The clinic started ART services in 2009 under a USAID-funded project that enabled the clinic secure ART site accreditation with the Uganda government. This enabled the clinic to receive regular supplies of free ART commodities from the national medicines supplier. A number of patients active on ART were 19. This clinic offers adult ART services.

Table 3 shows that out of a maximum score of 32 for the institutionalization of ART programs, the two highest-ranked cases (P-001 and PNFP-001) scored 26.8 and 26, respectively. The two lowest ranked health facilities (P-002 and PFP-001) scored 3.9 and 2.7, respectively.

The two health facilities with the highest scores for the institutionalization of ART programs were at a higher level of care in the Ugandan health system [26] compared to those with the lowest scores. P-001 and PNFP-001 are categorized as hospitals whereas, P-002 and PFP-001 are health centers. Additionally, there were variations in ART patient volumes across the cases with the highest scoring cases (P-001 and PNFP-002) reporting significantly higher patient volumes compared to P002 and PFP-001.

Table 3 reveals that the two cases with the highest scores for ART institutionalization had significantly more staff assigned to the ART clinic compared to the two health facilities with the lowest scores for ART institutionalization.

The ART program leaders at P-001 and PNFP-001 had more advanced academic training compared to the program leaders at P-002 and PFP-001. With regard to setting, the highest scoring facilities were are all based in urban settings compared to the lowest scoring facilities which were located in rural settings or urbanized parts of rural areas.

Table 3 shows that the ART clinic at P-001 and PNFP-001 run on 3 and 5 days of the week, respectively. On the other hand, the ART clinic run on one day of the week at P-002. PFP-001 did not have a designated ART clinic day.

### Section B

This section presents selected responses to questionnaire items from the Level of Institutionalization (LoIn) Scales with respect to the four case-study facilities (Table 4).

**Table 3.** Characteristics of case-study health facilities.

Cases acronym	P-001	PNFP-001	P-002	PFP-001
Ownership-type	Public	Private Not-for-profit	Public	Private for-profit
Level of care	Regional Referral hospital	General Hospital	Health centre IV	Health centre III
Setting	Urban	Urban	Rural	Peri-urban
Highest level of education of ART program leader	Masters’ degree	Masters’ degree	Diploma	Bachelor’s degree
Institutionalization Score (out of 32)	26.8	26	3.9	2.7
ART scale-up commencement	2004	2005	2006	2009
ART clinic patient load	24,408	4,337	458	19
ART Clinic staffing strength	43	53	02	01
ART Clinic day frequency *(Number of days per week)*	3	5	1	No designated ART clinic day

**Table 4.** Level of Institutionalization (LoIn) questionnaire responses.

	P-001	PNFP-001	PFP-001	P-002
***Production***				
Does your health facility have a strategic plan in which it plans to continue providing ART for at least five years or more?	Yes	Yes	No	No
Have any manuals or procedures for ART delivery at your site been put in writing?	Yes	Yes	No	No
Has a schedule (e.g. timetable) been used for implementing program activities been put in writing?	Yes	Yes	No	No
What is your best estimate of the proportion of the ART program as originally designed at the start has since been adapted to fit your health facility’s context?	Most Aspects	Most aspects	No aspects	Few aspects
What is your best estimate of the number of staff involved with the ART program who have written job descriptions?	Most	Most	None	None
***Managerial***				
Has the ART site manager been formally appointed with a formal contract?	Yes	Yes	No	No
Have formal job descriptions been written for staff involved in the ART program?	Yes	Yes	No	No
Is there a staff member at your site who you would regard as ART program champion?	Yes	Yes	No	No
***Maintenance***				
Do any of the ART program staff hold permanent positions?	Yes	Yes	No	Yes
Do other staff at your facility, other than ART program staff, actively contribute to the program’s operations?	Yes	Yes	No	Yes
***Supportive***				
Has the ART program made a transition from trial or pilot status to permanent status?	Yes	Yes	No	No
Has the program been assigned permanent physical space within your organization?	Yes	Yes	No	Yes
What is your best estimate of how permanent the funding is for salaries of the staff most closely associated with the ART program?	Minimally permanent	Not at all	Not at all	Not at all

Items are selected from each of the four domains or ‘sub-systems’ that are theorized to make up a health facility [28].

#### Production

Table 2 shows that the two cases with the highest scores for the institutionalization of ART programs (P-001 and PFP-001) had written procedures and manuals for ART service delivery. In contrast, the two cases with the lowest scores for ART institutionalization (P-002 and PFP-001) reported that ART delivery procedures had not been put in writing.

Table 2 further shows that the two health facilities with the highest scores for institutionalization had organizational strategic plans which spelt out the sustainment of ART service delivery as a goal for the next five years. The cases with the lowest scores indicated that they did not have an organizational strategic plan in place.

The cases with the highest scores for ART institutionalization indicated that they had made adaptations to ART service delivery models to improve fit with their operational context. In contrast, P-002 and PFP-001 indicated they had not made any adaptations to ART interventions.

#### Managerial

The cases with the highest scores of the institutionalization of ART programs (P-001 and PNFP-001) reported that health workers at their ART clinics (including their supervisors) had been formally appointed in their roles. On the other hand, (P-002 and PFP-001) indicated that personnel in their ART clinic had not been formally appointed. The quantitative data, in this regard, were triangulated with qualitative interviews where one of the cases (PFP-001), a for-profit Clinic, indicated that all the health workers in the clinic were designated as temporary staff. In contrast, personnel at P-001 and PNFP-001 reported that several salaries of personnel in the ART clinics were drawn from project grants and had that formal contracts has been signed based on this funding.

The presence of a ‘program champion’ for ART programs differentiated between the two categories of cases. P-001 and PNFP-001 both indicated the presence of an internal program champion. In contrast, P-002 and PFP-001 indicated that they did not have a staff member in their health facilities whom they regarded as a program champion.

#### Maintenance

Data generated from the Level of Institutionalization Scales (Table 2) shows that P-001 and PFP-001, reported that the ART program received service support from staff from other sections or departments of their health facilities. Only one of the other two cases (P-002), a public health centre IV, indicated that staff from other departments within the health facility occasionally shared the workload with personnel in the ART clinic.

#### Supportive

When asked whether the ART program had transitioned from a pilot to a more ‘permanent’ status, the cases with highest scores for the institutionalization of ART programs indicated ‘Yes’ (Table 2). This was in contrast to the other two cases which selected ‘No’.

These quantitative data were triangulated with qualitative data reported in Section C, which were consistent in demonstrating significant differences in the extent of ‘embeddedness’ of the ART program in organizational routines across the two categories of cases.

### Section C

This section presents results of the qualitative interviews with representatives from the four health facilities to understand the reasons for variations in scores for the institutionalization of ART programs between the two categories of cases.

#### Longer implementation history

An attribute that differentiated between the two categories of cases was the length and experience in implementing HIV interventions. P-001 and PNFP-001 both indicated that their earliest HIV intervention implementation history dates back to 1998 when both facilities participated in a pilot drug-access initiative in Uganda. Representatives from both cases indicated that they had accumulated considerable experience in implementing HIV interventions and that the delivery procedures had become routine.

*’We began treating patients with HIV here in 1998 even before ART was rolled out by the Uganda government and PEPFAR in 2005. We had an American researcher who was doing routine research here. He was heart-broken by the number of patients who needed treatment but couldn’t get it. When he returned home he secured support to start treating a few patients here.’ [Interviewee 2, P-001]*

In the qualitative interviews with representatives from P-002 and PFP-001, it was indicated that their earliest HIV implementation history was in 2006 and 2009, respectively. The latter cases commenced anti-retroviral therapy during the externally supported national emergency ART roll-out phase in Uganda.

The difference in HIV implementation history between the two categories of cases ranged between 8 and 11 years.

#### The role of staff training in the institutionalization of ART programs

A theme that emerged strongly across both P-001 and PNFP-001 was the importance of staff training in institutionalizing ART delivery procedures. Interviews with representatives of both cases revealed that they deliberately targeted mid-cadre especially nurses for trainings in ART management as a strategy of retaining ART delivery skills within their institutions. Mid-cadre, particularly nurses, were perceived as more stable at stations compared to medical doctors who often opted for further training and alternative career opportunities.

*‘Training of the health workers is very key. This means that these skills remain with the staff and even if donor funding ends or a project closes these practical skills remain with our staff. We deliberately focus on the training of especially nurses because those tend to stick around for years unlike young doctors who frequently opt for further training and leave when better opportunities arise’ [Interviewee 1, PNFP-001].*

The trainings in ART management were organized internally such as weekly continuing medical education (CME) sessions at PNFP-001 but also through invitations for attending off-site training workshops organized by the Uganda Ministry of Health or donor-funded implementing agencies. P-002 and PFP-001 indicated that several of their staff had attended trainings in ART management although many of them had been lost through attrition and staff transfers. A USAID-funded evaluation report from 2013 relating to HIV services delivery standards found that with respect to P-002, ART clinic workforce from this health facility, who were interviewed, indicated that they had not received training ‘with technical updates on essential topics such as adult and pediatric ART and the management of opportunistic infections’.

#### Retention of ART-proficient personnel

The retention of health workers who had been trained in ART management and had accumulated program experience in ART service delivery emerged as a distinguishing feature between the two categories of cases. Health facilities with the highest scores for the institutionalization of ART programs reported deliberate strategies for retention of ART-proficient health workers. These included securing sustained grants from funders for ART delivery that had project-based salaries for a number of their ART-proficient staff. At P-001, the majority of personnel in the clinic had served for not less than 5 years due to sustained external grants for ART delivery. Representatives of PNFP-001, a mission hospital, had longer terms of service among the four health facilities. On average, personnel in the ART clinic had served for at least 8 years. Conversely, the turnover of health workers trained in ART was reported as a barrier to the institutionalization of ART programs in the two health facilities with the lowest scores of institutionalization.

*’ Out of the four staff that our donor trained in managing ART, three have since resigned. This is not a big private clinic hence we are unable to give our staff competitive salaries. Whenever they receive better Job offers, they leave. That means they go with the skills in managing ART that they received here. So, you have to keep training new staff afresh all the time’ [Interviewee 3, PFP-001].*

At P-002, a public health centre IV, frequent transfers of ART-proficient personnel was reported as an impediment to the institutionalization of ART programs. At PFP-001, a for-profit clinic, a deliberate policy of not offering formal terms of service for all health workers in the clinic was acknowledged as a dis-incentive to long-term commitment by health workers.

#### Simplified manuals on ART delivery procedures

The diffusion of ART interventions through the development of simplified in-house ART service delivery manuals was reported as a facilitator to intervention institutionalization at both P-001 and PNFP-001.

*‘We developed our own manuals based on the national ART guidelines. All we do is simplify the language for our nurses and make the manuals more user-friendly for the staff’ [Interviewee 3, PNFP-001].*

During the on-site visits to P-001 and PNFP-001, evidence was seen of ART service delivery manuals and procedures laid out to ART clinic staff in form of posters pinned on staff noticeboards within the clinics. This included pictorial illustrations of the class of ARV drugs applicable to patients at varied disease stages. In contrast, investigator on-site visits to P-002 and PFP-001 did not find similar evidence. This finding was corroborated by a USAID-funded evaluation report of 2013 assessing Ugandan health facilities’ performance in HIV standard of care which found that with regard to P-002, ‘there was an absence of appropriate guidelines for ART and essential items for HIV care on the day of assessment’. Evidence from on-site observation notes were consistent with this finding with respect to the two health facilities with the lowest scores for the institutionalization of ART programs.

The finding that in-house manuals on ART delivery had been developed at PNFP-001 was corroborated with findings from the LoIn instrument which revealed that some aspects of the ART program were adapted to suit the operational context of this health facility.

#### Variations in level of donor support for ART programs

Insufficient support for ART scale-up implementation was frequently cited as a barrier to the institutionalization of ART programs at PFP-001 and P-002 (a for-profit clinic and a sub-district health facility respectively). Although all four participating health facilities received a minimum package of external support that included a free supply of anti-retroviral drugs, health worker training in ART management, on-site support supervision and ART program data support between 2004 and 2009, it emerged that there were slight variations in the scale of donor support received. A key distinguishing feature between the two categories of cases was salary support for key personnel in the ART clinic. The two highest scoring health facilities, a regional referral hospital and a large mission hospital, both indicated that a number of the health workers in their ART clinics drew salaries from the projects supporting ART scale-up implementation. This was not the case in PFP-001 and P-002, a for-profit clinic and sub-district public facility respectively. Project salaries can be twice as much as those paid in the public facilities [22].

*‘We have lost many of our staff who were trained in ART by our funder. The challenges of human resources are the same here as in other private health facilities. Unlike those large hospitals whom donors favour with project salaries, health workers here don’t get any salary support’ [Interviewee 1, PFP-001].*

*‘I only have two people to handle all those clients waiting outside, a clinical officer and psychiatric nurse. The rest are volunteers who are not medical people. Human resource is a big challenge here. If there was support I would request for from donors, number one is manpower’ [Interviewee 1, P-002].*

Interviews with representatives from the health facilities revealed that each of the four health facilities had a different ‘implementing partner’. However, all four were PEPFAR intermediary organizations through which donor support was channeled.

## Discussion

Many previous studies focused on the initial phase of ART scale-up implementation in Sub-Saharan Africa. This study was prompted by a dearth of systematic analysis of the level of institutionalization of ART programs in externally supported health facilities in Uganda. Our case-study analysis reveals wide variations in the degree of institutionalization of ART programs in health facilities in Uganda. In the two highest-ranked hospitals, ART interventions were highly integrated in the four sub-systems theorized to make up a health service organization. ART service delivery metamorphosed into standard procedure or a part of ‘normal’ organizational routine. The findings demonstrate that in these two hospitals, ART interventions have transitioned from a pilot state to a more ‘permanent’ state. Conversely, in the two health facilities with the lowest scores for institutionalization, ART interventions were still in ‘pilot’ mode and were still offered in an ad-hoc manner. ART service delivery had not yet transitioned into being an integral part of their organizations. The notion of intervention ‘permanence’ as an indicator of the program sustainability of innovations in health service organizations is consistent in the literature [14,16,34]. O’Loughlin et al [35], used perceived permanence of an intervention as an outcome measure in their investigation of the sustainability of heart health promotion interventions in Canada.

We found that the two health facilities with the highest scores for program institutionalization had a longer HIV intervention implementation history of between 8 and 11 years compared to the two health facilities with the lowest scores. The literature is relatively silent on the influence of intervention implementation experience on program sustainability outcomes. A notable exception is a 2001 study in the USA by Goodson and colleagues [19] which found that intervention implementation experience of heart health prevention services was a predictor of program institutionalization in host agencies. Although studies have been conducted on intervention institutionalization focusing on diverse content fields such as heart health [19], nursing [17,33], and health promotion [28], the unique contribution of this study is in applying these theoretical constructs to donor-funded ART programs in health facilities in Uganda. Moreover, the majority of studies conducted on the institutionalization of interventions have been from high-income contexts particularly from the USA and Canada [17–20,33]. This study adds to the limited evidence base on the concept of institutionalization of evidence-based interventions in resource-limited settings.

### Organizational context influences on the institutionalization of ART programs

Our analysis fills a void in the literature by contributing to our understanding of the organizational factors influencing the institutionalization of anti-retroviral therapy (ART) programs in Uganda. Our study reveals significant differences in the organizational contexts of the two categories of health facilities with variations in the level of institutionalization of ART programs. We found that the two health facilities with the highest scores for institutionalization were at a higher level of care in the Ugandan health system (referral hospitals) [37] compared to the two lowest scoring facilities which were lower-level health facilities. These data suggest that interventions aimed at deepening the institutionalization of ART programs especially at lower-level health facilities, which constitute the majority of health facilities in Uganda, are critical to long-term program sustainability [38]. This will necessitate empowering and strengthening the ART monitoring unit of Uganda’s Ministry of Health to deliver on its own mandate of monitoring national ART service delivery including supporting further research to explore the relationship between level of care and institutionalization outcomes of ART programs with a larger sample of health facilities.

In this study, we found that the presence of an internal ‘program champion’ for ART programs in participating health facilities was an attribute that distinguished between the two categories of cases. There was an absence of program champions for ART programs in the two health facilities with the lowest scores for degree of institutionalization of ART programs. The literature suggests that the presence of an internal champion, who is strategically placed to advocate for the needs of a program within an implementing agency, increases the likelihood of program continuation [14,35,37].

This study adds to evidence suggesting that factors internal to the organizational setting of an implementing agency are influential on program sustainability. There is support in the literature for the notion that differences in organizational capacity account for variations in program sustainability outcomes [39–41]. In an earlier seminal review, Shediac-Rizkallah and Bone [34] found that ‘’implementing organizations which were stable and mature were more likely to promote program institutionalization by providing a strong organizational base for new programs ‘‘. Glisson and colleagues [42] found that differences in the internal organizational culture and climate of the host implementing agency accounted for variations in the program sustainability outcomes of mental health interventions implemented in 200 clinics in the USA.

### Human resources for health implications of the study

A major contribution of this study is in utilizing Levels of Institutionalization (LoIn) Scales to systematically identify human resources for health as ‘the weak link’ within the elements theorized to make up a health service organization by Goodman et al. [28]. Our study unearthed significant differences in human resources for health attributes in the case-study health facilities. The two highest scoring hospitals had written job descriptions and formal terms of service for personnel in their ART clinics compared to the two health facilities with the lowest scores which indicated this was not the case. It became clear that health workers in ART clinics in the lowest scoring health facilities were without formal terms of service. Although the turnover of ART-proficient health workers was cited independently as a barrier to the institutionalization of ART programs in the latter health facilities, it is plausible that this could be related to the lack of formal terms of service for health workers in the two lowest-ranked health facilities which could be a disincentive to long-term commitment. In this respect, this study adds to the growing evidence suggesting that there are dynamic interactions in the factors influencing intervention sustainability [19,43–46]. This approach challenges linear and reductionist approaches employed in investigating bottlenecks to the scale-up of public health interventions [44].

Our study brings into critical focus the challenges of health worker retention in Uganda in the context of unattractive monetary reward systems, especially in the public sector, and the need for devising non-monetary incentives [22,47]. Studies conducted in Uganda show that beyond monetary rewards, health workers are motivated by the desire to serve rural folk in their home districts where they can leverage their social capital as well as tend to food gardens to supplement their meager salaries [47]. Several studies highlight the role of leadership styles in motivating the workforce [17,18,22,33].

Our findings demonstrate that health facilities deliberately targeted mid-cadre particularly nurses for health worker trainings in ART management as a strategy of promoting the institutionalization of ART programs in their health facilities. Although previous studies have highlighted task shifting to non-physicians as a response to the shortage of physicians in comparison to the relative availability of mid-cadre such as nurses [22,48,49,50], this study suggests that providers strategically targeted mid and lower cadre health workers because they were perceived as less likely to leave their posts for ‘greener pastures’ or for more advanced training as compared to physicians. Targeting mid and lower-cadre for trainings in ART management as a strategy for the long-term institutionalization of ART programs in resource-limited settings on account of their relative ‘longevity’ at duty stations is worthy of further research. In this connection, reviews of the national curriculum for physicians and non-physician cadre at tertiary and non-tertiary institutions in high-burden countries is recommended in order to strengthen training in the clinical management of HIV which would undoubtedly boost skills in ART-proficiency in the graduating workforce.

### Implications for the sustainability of ART programs in resource-limited settings

The highest-ranked health facilities associated the institutionalization of ART programs with sustained staff trainings in ART management, the retention of ART-trained personnel especially nurses, the diffusion of ART interventions through written ART delivery procedures, simplified in-house manuals and pinning ART delivery procedures on staff notice boards.

These findings are relevant to funders and program managers of ART programs in Uganda and other resource-limited settings in as far as they provide guidance on how to deepen the institutionalization of ART interventions in host organizations. This is especially topical in the context of declining international assistance for ART scale-up, amidst the escalating demand for antiretroviral therapy following the adoption of WHO’s universal ‘test and treat’ policy [20]. The role of training in fostering the sustainability of diverse interventions in implementing organizations is supported in the literature [34,37,51,52].

An important contribution of this case-study analysis is in demonstrating that a one-size-fits-all approach to the sustainability of ART scale-up implementation in resource-limited settings may miss nuances in the organizational contexts of implementing health facilities. Our findings demonstrate that well-established, large, urban-based hospitals have had more success in institutionalizing ART interventions and could have a higher chance of sustaining these interventions compared to lower-level, rural health facilities in a post-donor scenario. Interventions aimed at deepening the institutionalization of ART programs in the latter category of health facilities such as through prioritizing workforce training in ART management, devising retention strategies for ART-proficient personnel, encouraging the development of simplified manuals for ART delivery and pinning posters of ART delivery procedures on staff noticeboards could promote long-term ART program sustainability in Uganda and other resource-limited settings.

Our findings demonstrate that relative to large hospitals, small for-profit clinics and lower-tier health centers have a weak internal capacity for ART service delivery partly due to inability to attract and retain health workers. This will likely continue to be a fundamental barrier to attaining universal ‘test and treat’ targets in Uganda where almost a half of all health facilities are small private health centers [53]. Studies in Uganda have documented the limited capacity of small for-profit clinics for patient follow-up, maintaining basic ART program records, having smaller HIV client loads due to a ‘narrower HIV services menu’ and the overall tendency of patients to gravitate toward higher-tier hospitals in seeking HIV care [33,53–55].

With respect to the influence of human resources for health constraints, on the attainment of ART scale-up targets in resource-limited settings, Beisma and colleagues [56], in a study in Zambia and Mozambique, highlighted the importance of addressing human resources for health constraints which often receive inadequate attention from donors relative to other individual building blocks of the health system such as financing and ART medicines [57]. The importance of addressing human resources for health constraints in order to attain ART scale-up targets in resource-limited settings is consistent in the literature [22,49,58]. However, there are mounting calls for funders of public health interventions to adopt ‘whole-of-system’ approaches which utilize ‘systems thinking’ as opposed to fragmented approaches which focus on individual building blocks of the health system [25,44,45,59].

In this study, we found that there were variations in the level of donor support received for ART service delivery in the four case-study facilities. Representatives from the two facilities with the lowest scores for institutionalization perceived the level of support received from donors for their ART programs to have been less than that received by higher-tier hospitals. It is plausible that having significantly higher patient volumes and the referral character of the latter category of health facilities could have advantaged them in the scale of donor support received. In this regard, Bowman et al [43], earlier proposed that ‘’the longevity of an adopted intervention may be a direct function of original implementation intensity’’ and that variations in the implementation ‘dose received’ should be factored in *post-hoc* analyses of long-term program sustainability. The findings of this study offer some empirical credence to this notion.

#### Non-donor HIV financing mechanisms in Uganda

A related study in Uganda [10], documents the increasing diversification of HIV funding away from the traditional sources of funding from PEPFAR and The Global Fund through alternative financing mechanisms. This study [10] highlights the differentiated prospects for the long-term sustainability of ART programs by the ownership-type of a health facility in Uganda. For-profit health facilities are shown to offer more promise for non-donor HIV financing exemplified in innovations such as special ‘HIV’ medical insurance schemes, revenue windfalls from selling brand ARV drugs to upper middle-class Ugandans, private employee medical insurance coverage expansion, after-hours executive ‘VIP’ clinics that support poorer patients [60,61]. However, this same study reveals that not for-profit health facilities are more donor-dependent and would likely be more impacted by cuts in donor funding. With respect to public facilities, the planned introduction of a national HIV tax in Uganda to support an AIDS Trust Fund (ATF) holds the promise of a significant reduction in dependence on external donor funding through a planned levy on soft drinks that would boost reliance on domestic sources for the national HIV response [10].

In recent years, the national HIV response in Uganda has been dominated by a focus on HIV treatment coverage expansion, combined approaches to that engender HIV prevention need to be scale-up. This would entail addressing the key drivers of the epidemic in Uganda including its socio-cultural determinants, thereby reducing HIV incidence which in turn would reduce the imperative for international assistance for HIV assistance responses.

#### Further research

Whereas our study compares the level of institutionalization of ART programs at two levels of care (tertiary/primary) in the health system in Uganda, a recommendation for further research is to conduct a comparative case-study of level of institutionalization of ART programs among health facilities at the same level of care (tertiary, secondary, primary) and size to reduce variability in the sample or to rank health facilities into quartiles based on their scores for the institutionalization for ART programs and to compare cases within quartiles.

### Limitations

Although this study offers an in-depth, contextualized understanding of the factors impacting the institutionalization of externally supported ART programs at higher and lower levels of care of the Ugandan health system, the findings need to be interpreted within the context of the study’s limitations. The small sample size of four health facilities detracts from the statistical generalization of the study findings although the theoretical transferability of the study findings is possible. The use of self-report data such as a self-administered questionnaire rendered the study liable to social desirability bias. This was mitigated by securing at least three informants per health facility to compare interviewee data and triangulation with different sources including document review and questionnaire data. The strengths of this study include a contribution to the sparse evidence on the long-term institutionalization of ART programs in health facilities in Uganda, the adoption of processes for ensuring rigor in case-study analysis, the use of a multi-methods approach and a longer-term lens on ART scale-up implementation in health facilities in Uganda as well as a contribution to the limited evidence base on health program sustainability in resource-limited settings.

### Conclusion

There were wide variations in the degree of the institutionalization of ART programs in the two categories of case-study health facilities. Whereas the two highest scoring health facilities had integrated ART into ‘normal’ or regular organizational routines, in the two lowest scoring health facilities, ART interventions were still in a ‘pilot’ mode. Together, the four cases suggest that the level of institutionalization of ART programs is differentiated by level of care in the Ugandan health system. Further research exploring the relationship between level of care of a health facility and degree of institutionalization of ART programs is recommended. Interventions aimed at deepening the institutionalization of ART programs in lower-tier health centers at the level of human resources for health (workforce contracts for ART-proficient personnel, staff retention strategies, strengthening clinical HIV management training in pre-service institutions) and programming (in-house simplified manuals, regular trainings in ART) could enhance the long-term sustainability of ART programs.

## Paper context

Although numerous studies have documented the pilot phase of ART scale-up in health facilities in Uganda, especially from a clinical perspective of patient outcomes, there is a dearth of evidence on long-term program institutionalization outcomes. This study highlights the wide disparities in the level of institutionalization of ART programs in health facilities in Uganda and proposes remedial measures for deepening program institutionalization especially in lower-level health facilities.
